# Clinical and Biochemical Implications of Hyaluronic Acid in Musculoskeletal Rehabilitation: A Comprehensive Review

**DOI:** 10.3390/jpm13121647

**Published:** 2023-11-26

**Authors:** Giorgia Natalia Iaconisi, Nunzia Gallo, Laura Caforio, Vincenzo Ricci, Giuseppe Fiermonte, Simone Della Tommasa, Andrea Bernetti, Vincenza Dolce, Giacomo Farì, Loredana Capobianco

**Affiliations:** 1 Department of Biological and Environmental Science and Technologies (Di.S.Te.B.A.), University of Salento, 73100 Lecce, Italy; giorgianatalia.iaconisi@unisalento.it (G.N.I.); andrea.bernetti@unisalento.it (A.B.); 2 Department of Engineering for Innovation, University of Salento, 73100 Lecce, Italy; nunzia.gallo@unisalento.it; 3 Department of Basic Medical Sciences, Neurosciences and Sense Organs, Aldo Moro University, 70121 Bari, Italy; laura.caforio@uniba.it; 4 Physical and Rehabilitation Medicine Unit, Luigi Sacco University Hospital, ASST Fatebenefratelli-Sacco, 20157 Milan, Italy; vincenzo.ricci58@gmail.com; 5 Department of Biosciences, Biotechnologies and Biopharmaceutics, University of Bari, 70125 Bari, Italy; giuseppe.fiermonte@uniba.it; 6 Department for Horses, Faculty of Veterinary Medicine, Leipzig University, 04109 Leipzig, Germany; della.tommasa@vetmed.uni-leipzig.de; 7 Department of Pharmacy, Health and Nutritional Sciences, University of Calabria, 87036 Arcavacata di Rende, Italy; vincenza.dolce@unical.it; 8 Department of Translational Biomedicine and Neuroscience (DiBraiN), Aldo Moro University, 70121 Bari, Italy

**Keywords:** hyaluronic acid, active biomolecule, biomaterial, rehabilitation, HA injection, physical therapy

## Abstract

Hyaluronic acid (HA) naturally occurs as a biopolymer in the human body, primarily in connective tissues like joints and skin. Functioning as a vital element of synovial fluid, it lubricates joints, facilitating fluid movement and diminishing bone friction to protect articular well-being. Its distinctive attributes encompass notable viscosity and water retention capacities, ensuring flexibility and absorbing shock during motion. Furthermore, HA has gained significant attention for its potential benefits in various medical applications, including rehabilitation. Ongoing research explores its properties and functions, especially its biomedical applications in several clinical trials, with a focus on its role in improving rehabilitation outcomes. But the clinical and biochemical implications of HA in musculoskeletal rehabilitation have yet to be fully explored. This review thoroughly investigates the properties and functions of HA while highlighting its biomedical applications in different clinical trials, with a special emphasis on its role in rehabilitation. The presented findings provide evidence that HA, as a natural substance, enhances the outcomes of musculoskeletal rehabilitation through its exceptional mechanical and biochemical effects.

## 1. Introduction

Hyaluronic acid (HA) is a naturally occurring heteropolysaccharide with a high molecular weight (MW), lacking protein and sulfated components. It has exceptional biocompatibility, biodegradability, and nonimmunogenic properties, making it widely used in therapeutic and cosmetic domains [1,2].

It plays a crucial role in various biological processes, including wound healing and bone regeneration [3]. Additionally, HA acts as a regulator of fundamental cellular functions such as adhesion, proliferation, and differentiation [3]. Functioning as a biomaterial, it is actively produced by specific cell types like type B synoviocytes, fibroblasts, and chondrocytes [4]. Its functions encompass improving tissue hydration and firmness, aiding wound healing, controlling inflammation, and providing effective lubrication [1,2].

The U.S. Food and Drug Administration (FDA) has recently proposed reclassifying HA-based compounds, devices, products, and delivery systems as therapeutic drugs. This reclassification is based on scientific evidence, particularly highlighting HA’s pain-relieving properties, especially in cases of joint inflammation. This proposal stems from HA’s demonstrated biochemical effects in living systems [5,6,7].

Due to its versatile properties, the global HA market is projected to reach approximately USD 15.4 billion by 2025. This growth is driven by factors like the increasing elderly population and recognition of the natural substances’ benefits in various products [2,8].

HA-based dermal fillers lead the market, known for their antiaging and facial contouring properties [9]. HA is also used in topical cosmetic formulations, offering substantial advantages for skin hydration [10].

In the medical field, HA-based products have shown efficacy in treating severe conditions like osteoarthritis (OA). High MW injectable HA is commonly used to alleviate pain and enhance joint mobility, often in combination with other treatments [11,12,13,14,15,16,17,18].

HA significantly promotes joint health [19,20], reduces pain [21], aids in injury recovery [22], and supports rehabilitation therapy [12]. In vitro studies reveal that HA effectively enhances the mechanical properties of synovial fluid and biochemically regulates joint tissues [20,23]. As a result, it is commonly used in local joint injections to reduce pain and enhance functionality [19,24].

In addition, HA is used in several other fields, from drug delivery systems to infection treatments [2]. In fact, it can also be combined with antibacterial treatments based on zinc, zinc oxide, or silver and copper [25,26].

However, HA-based products encounter challenges due to high production costs. HA can be derived from animal tissues or produced through bacterial synthesis. The purification process is critical to eliminate contaminants like proteins and endotoxins that can induce inflammatory responses even at low concentrations [27,28]. Furthermore, the presence of other GAGs can present a notable challenge. This becomes particularly evident when extracting HA from animal tissues, as the extraction process may lead to the simultaneous extraction of additional GAGs, including chondroitin sulfate. Consequently, ensuring the purity of the final product is imperative to attain HA of superior quality [29]. Unfortunately, the production expenses, ranging from $1000 to $2000 per kilogram for cosmetic-grade HA and $40,000 to $60,000 per kilogram for medical-grade HA, make these products inaccessible to some customers and patients [30].

Considering this, research is focused on optimizing HA production methods to obtain a high-quality product at affordable costs [2].

The literature extensively explores the applications of HA, yet there exists persistent confusion surrounding its clinical and biochemical functions, as well as its efficacy in rehabilitating musculoskeletal pathologies. After providing an in-depth overview of HA’s structure, functions, and properties, this review shifts its focus toward a detailed examination of its current applications. Notably, it underscores HA’s pivotal role as a bioactive molecule in combination with rehabilitation for the treatment of musculoskeletal diseases. The review meticulously analyzes both biochemical and clinical implications, investigating advancements in this field over the past decade. This comprehensive approach aims to bring clarity and organization to the abundant scientific evidence found in recent literature.

To achieve this goal, an extensive search was conducted on studies exploring the current status and the role of HA as a therapeutic agent in combination with physical and rehabilitative therapy for musculoskeletal pathologies. The electronic search engines used were PubMed (https://pubmed.ncbi.nlm.nih.gov, accessed on 5 September 2023), ScienceDirect (https://www.sciencedirect.com, accessed on 5 September 2023), Google Scholar (https://scholar.google.com, accessed on 25 September 2023), and U.S. National Library of Medicine (https://clinicaltrials.gov, accessed on 25 September 2023).

**Figure 1:**
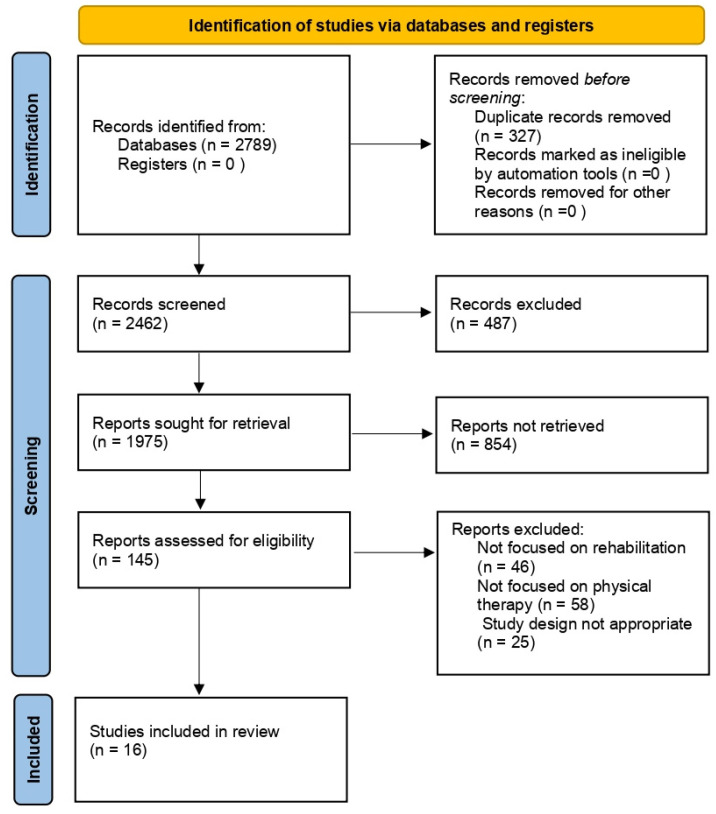
Flow chart of the analyzed studies.

The keywords used were ‘hyaluronic acid’, ‘rehabilitation therapy’, and ‘therapeutic molecule’. Several synonyms were searched for each keyword (i.e., hyaluronan, hyaluronate, physical therapy, therapeutic exercise, physical therapy). The search included the recent updates (2013–2023) related to HA and rehabilitation (including clinical trials as well as in vitro and in vivo studies) independently from their level of evidence (Figure 1).

## 2. HA: Biochemical Structure, Functions, and Properties

**Figure 2:**
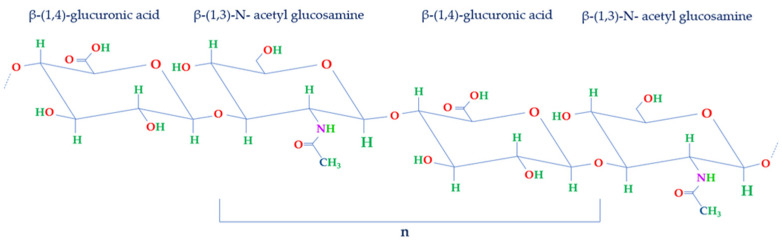
Structure of disaccharide repeating unit of HA. HA structure is made of repeated units of NAG and GlcA held together by β-1,3 and β-1,4 glycosidic bonds (n).

Comprising a glycosaminoglycan (GAG), HA consists of a repetitive arrangement of disaccharide units (n). These units are structured with β-1,4-glucuronic acid (GlcA) and β-1,3-N-acetyl glucosamine (NAG), interconnected by means of β-1,3 and β-1,4 glycosidic linkages (Figure 2) [1,2].

At physiological pH, HA exists in anionic, ionized, or salt form, such as sodium hyaluronate. In this condition, the molecule carries negative electrostatic charges due to the presence of dissociated carboxylic groups on each monomer; hence, the molecule shows anionic properties, making it very hydrophilic.

Indeed, these negative electrostatic charges play a significant role in its interactions with both water and other molecules within the body. As a result, HA is renowned for its remarkable ability to retain water, which contributes substantially to the maintenance of skin elasticity and hydration [1,2].

Within biological systems, the mentioned repeating disaccharides have the potential to combine into polymeric networks exceeding 10,000 units, resulting in high MW structures surpassing 4 megadaltons (MDa) [1,31]. This characteristic mesh-like structure serves as a hindrance to different external substances, such as bacteria and infectious agents, thereby reducing their filtration capacity [32].

The scientific evidence from the literature suggests that the average length of individual disaccharides in the biopolymer HA is approximately 1 nm [33]. For instance, an HA polymer with 10,000 repetitions could extend to around 10 µm. These HA chains within the cellular matrix form a dense brush-like structure known as the pericellular matrix (PCM), to which various proteoglycans attach and modify the structure of the HA chains. The composition and dimensions of the PCM layer vary from tissue to tissue based on their biological functionality [5].

It is important to highlight that the biological function of this polymer relies on its MW. HA is most found in a high MW form, with sizes generally exceeding 1000 kDa. In this state, it possesses biophysical qualities that serve as a lubricant. However, high MW HA can be broken down in vivo by hyaluronidases, a group of enzymes that break its chains, resulting in smaller (<250 kDa) and intermediate-sized (250–1000 kDa) fragments [34]. If the processes of synthesis and degradation are not appropriately balanced, the biochemical and rheological characteristics of the extracellular matrix, where HA is a primary component, can be altered. This, in turn, can contribute to the development of various pathological conditions [34].

Indeed, HA plays a crucial role in maintaining joint health by interacting with cartilage components, particularly aggrecan [35]. Aggrecan, a proteoglycan of the articular cartilage’s extracellular matrix, provides resilience to compressive loads. Its dynamic structure undergoes constant changes due to synthetic and degradative processes. Within the extracellular matrix, aggrecan forms proteoglycan aggregates, contributing to the mechanical properties of articular cartilage [35].

Structural changes in aggrecan during synthesis affect sulfate chain composition, while degradative events, prevalent in OA cartilage, can lead to aggrecan depletion and cartilage erosion. In early OA stages, the enhancement of aggrecan production and the inhibition of its degradation may slow destructive processes, preserving cartilage integrity [35].

Aggrecan production is crucial in cartilage repair techniques involving stem cell or chondrocyte implantation, aiming to promote the synthesis of aggrecan for the repair and regeneration of damaged cartilage [35].

In summary, the interactions between HA and aggrecan are vital for joint maintenance. While changes in aggrecan structure can contribute to articular cartilage demise, interventions enhancing aggrecan production and inhibiting degradation may promote cartilage survival, especially in OA and cartilage repair [35].

In addition to its chemical and mechanical characteristics, HA engages with various receptors found on cell membranes and specific proteins called hyaladherins [31,36]. These interactions trigger specific signal transduction responses.

The primary receptors responsible for interacting with HA are the cluster of differentiation 44 receptor (CD44R) and the receptor for hyaluronan-mediated motility (RHAMM). These receptors play vital roles in processes such as cellular adhesion, proliferation, and migration [31,36,37]. RHAMM shares certain characteristics with CD44 and contributes to cellular motility [38].

In contrast, lymphatic vessel endothelial hyaluronan receptor 1 (LYVE-1) serves as the receptor exclusively for the lymphatic system’s interactions with HA. Meanwhile, the hyaluronan receptor for endocytosis (HARE) governs the endocytosis of HA. Notably, studies have indicated that fragments of HA can activate Toll-like receptor 2 (TLR-2) and Toll-like receptor 4 (TLR-4), resulting in an increased adhesion of monocytes and other immune cells. This, in turn, influences the modulation of the inflammatory response [39].

**Figure 3:**
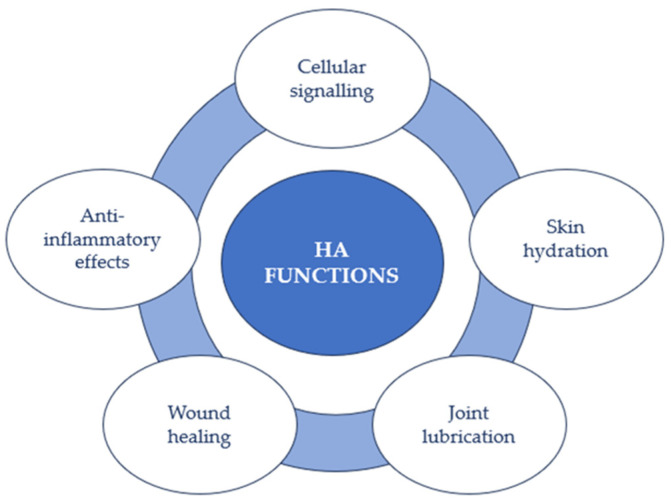
HA functions.

In this way, HA participates in numerous biological and cellular processes (Figure 3). Indeed, HA can envelop cells with a pericellular coat and bind to cell receptors, influencing cell activities like proliferation, migration, and gene expression. Additionally, HA’s anti-inflammatory effects hinder the movement of specific immune cells and reduce inflammation, potentially aiding in managing knee pain after surgery [40]. Moreover, it possesses the ability to impact various cellular processes, encompassing cell migration, proliferation, and differentiation. This renders it significant in tissue development, restoration, and overall tissue well-being [1,2].

Furthermore, one of the most prevalent roles of HA is linked to skin hydration. Due to its aptitude for retaining water, HA aids in maintaining the skin’s suppleness, smoothness, and adequate hydration, which holds significance for a youthful and healthy appearance [2,31].

Simultaneously, within joints, HA acts as a lubricant and shock absorber due to its substantial presence in synovial fluid. This function diminishes friction between joint surfaces and provides a cushioning effect crucial for ensuring joint mobility and comfort [41]. In particular, HA has multiple important functions concerning cartilage’s trophic status and the intra-articular environment’s regulation [4,40,42]. Its distinct viscoelastic properties grant synovial fluid exceptional shock absorption and lubrication abilities, and its large size and hydrophilic nature retain fluid in the joint during movement [40].

Furthermore, it has been demonstrated that HA is beneficial in promoting the migration and proliferation of fibroblast cells. For these reasons, combining HA therapy with rehabilitation could offer advantages for intra-articular conditions [40].

HA also contributes to the wound-healing process by aiding in the regulation of inflammation and promoting the growth of new cells. This role is pivotal for the restoration of damaged tissues, and HA additionally assists in the creation of a matrix that supports tissue regeneration [43].

## 3. HA in the Rehabilitation of Musculoskeletal Diseases

**Figure 4:**
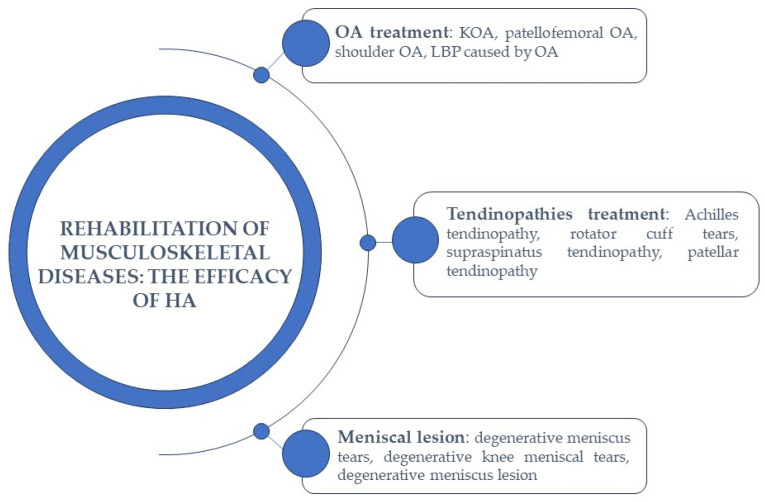
The efficacy of HA in the rehabilitation of musculoskeletal diseases.

The versatility of HA, its capacity to retain water, its compatibility with the human body, and its natural biodegradability all contribute to its widespread appeal in numerous therapeutic applications. These qualities continue to position HA as a highly effective therapeutic compound for addressing various medical conditions, including musculoskeletal diseases, and applications [44] (Figure 4). Consequently, a range of HA-based products have been developed and are readily available in the market. Furthermore, HA is not only employed on its own but also in conjunction with other bioproducts and therapies, further expanding its utility.

### 3.1. HA-Based Therapy in Osteoarthritis Treatment

As described above, HA is found in substantial amounts, particularly within cartilage and synovial fluid. It holds a vital function in the natural biomechanics of synovial fluid, governing tissue lubrication and elasticity [7,45].

The degradation of HA is a gradual process that can take place through enzymatic or nonenzymatic reactions. When HA undergoes degradation or its synthesis slows down, it leads to a decrease in its MW, which in turn affects various physical and chemical properties such as tissue volume, viscosity, and elasticity. HA is a molecule that is abundant in the knee joint, particularly in synovial fluid, but it is also present in articular cartilage [14].

It is crucial to emphasize that the decline in HA levels is not only linked to aging but also correlates with the progression of specific conditions, such as OA [7,45]. In this case, the viscoelastic properties of synovial fluid undergo substantial changes due to a reduction in both HA concentration and MW within the synovial fluid [18].

The pathogenic processes of OA induce modifications in the metabolism of type B synoviocytes, leading to the production of pathogenic HA [18,46]. This alteration in the quality of HA contributes to heightened mechanical strains within the joint, resulting in reduced lubrication capacity and, consequently, the stimulation of nociceptors that cause pain [47,48,49,50].

Notably, there exists a significant connection between joint movements and the active secretion of HA. The quantity of HA released appears to be influenced by both the frequency and duration of joint movements. This discovery suggests a biochemical mechanism that provides backing for the potential utilization of physical therapy in the management of OA [51,52,53].

Specifically, KOA emerges as a prevalent global joint condition, and its occurrence increases with age [13,54,55].

In particular, the knee joint presents a challenging biomechanical environment due to its avascular, aneural, and alymphatic nature. In this context, synovial fluid plays a crucial role as a lubricant with unique rheological properties [14]. Additionally, synovial fluid possesses the capability to scavenge free radicals and regulate intracellular activity and protein binding. The progression of KOA is intimately linked to the deterioration of synovial fluid’s lubricating function [14]. This deterioration results from the depolymerization of endogenous HA with high MW (ranging from 6500 to 10,900 kDa), transforming it into low MW HA (ranging from 2700 to 4500 kDa). HA with low MW leads to synovial fluid that exhibits significantly reduced mechanical and viscoelastic properties [14].

Moreover, treatment strategies for OA encompass not only surgical interventions but also efforts to reduce risk factors [56,57,58]. Additionally, they may involve participation in physical therapy and rehabilitation programs [12,59,60], as well as the use of oral corticosteroids or nonsteroidal anti-inflammatory drugs (NSAIDs), the use of oral dietary supplements [61,62], intra-articular injections of corticosteroids, HA, or platelet-rich concentrates [63,64,65,66,67,68,69,70,71,72,73].

Generally, the injection of high MW exogenous HA can be employed to mitigate the loss of synovial fluid properties caused by the endogenous depolymerization of natural HA. It is important to note that exogenous HA does not replace or restore the body’s own HA, but its presence in the joint induces symptom improvement in KOA over several months [74]. This improvement occurs because exogenous HA stimulates the synthesis of glycosaminoglycan and/or proteoglycan, allowing the synovial fluid to maintain its viscoelastic properties. Additionally, exogenous HA exhibits a significant anti-inflammatory effect, which, through secondary mechanisms, helps reduce pain.

FDA-approved HA injection products come with various physicochemical characteristics that can make one product more efficient or competitive than another. Among these characteristics, MW is a key consideration for clinicians, with options ranging from 500 up to 6000 kDa. Generally, the higher the MW, the longer the therapeutic efficacy [74].

Based on recent literature, exercise rehabilitation therapy continues to be a primary option for OA treatment [68,75]. Its crucial role lies in restoring muscle balance and proper load distribution, ultimately alleviating pain and enhancing function.

In particular, to assess pain in KOA, the most commonly used scales are the Visual Analog Scale (VAS) and the Western Ontario and McMaster Universities Osteoarthritis Index (WOMAC), in which the severity of pain is correlated with intra-articular changes. The radiological score Kellgren-Lawrence (KL) is associated with the presence of osteophytes and cartilage lesions and serves as an independent predictor for the VAS scale [76].

A recent meta-analysis of randomized controlled trials conducted by Liao et al. (2023) widely investigated the impact of combining intra-articular injections with rehabilitation therapy in patients suffering from KOA. Their specific emphasis was on assessing the combined therapy’s influence on pain levels, overall functional improvement, and walking capacity. Through this approach, they aimed to establish the most effective treatment option by ranking the effectiveness of each combined therapy regimen and exploring any factors that might influence treatment outcomes [77].

In the specific case of the combination of intra-articular injections of HA and physical therapy, it was observed that this treatment appeared to be more effective in reducing pain compared with physical therapy alone. Additionally, it allowed for better walking capacity in the short term, both compared with physical therapy alone and in comparison with other intra-articular injections [77].

In a blinded and randomized controlled study, Saccomanno et al. (2016) investigated the effectiveness of combining HA injections with personalized rehabilitation programs based on exercise-based rehabilitation (EBR) in patients with KOA [13]. A total of 165 participants with KOA were randomly assigned to three treatment groups: the first group received three intra-articular injections of high MW HA (Orthovisc 2 mL; 15 mg/mL; Anika Therapeutics Inc., Bedford, MA, USA); the second group underwent only rehabilitative exercises (detailed program of knee exercises); and the third group received both treatments, combining HA injections with an EBR program [13].

The analysis of the data revealed that all three treatment groups experienced decreased pain, improved flexibility, and enhanced functionality.

In particular, patients in group 1 showed the least pronounced treatment effect, which remained consistent over time. In contrast, patients in group 2 experienced notable declines in pain, stiffness, and functional results from the initial to the final follow-up. Finally, analysis of the WOMAC pain subscale revealed that patients in group 3 exhibited the most significant reduction in pain at the 1-month follow-up [13].

In a recent parallel randomized trial, Onu et al. (2022) conducted a study to evaluate the effectiveness of a HA-based product when used in combination with physical therapy for KOA treatment [15]. The research focused on a group of 52 patients who had been diagnosed with stage 2 KOA according to the KL scale, as determined through radiological examination of anterior-posterior knee X-rays [15]. In KL stage 2, patients typically exhibit osteophytes and narrowing of the joint space.

The patients were divided into two groups. The pilot group, consisting of 37 patients, received intra-articular HA injections (3 mL of Kombihylan, Ropharm, Romania) in combination with 10 consecutive sessions of physical therapy [15]. On the other hand, the control group was treated only with HA injections. In this case, a high-molecular-weight HA, 3 MDa, in the form of a viscoelastic solution obtained through bacterial fermentation of a Streptococcus strain, was used [15].

In particular, the physiotherapy treatment, in this case, involved several components: electrotherapy, specifically conventional transcutaneous electrical nerve stimulation (TENS) electroanalgesia; low-level laser therapy (LLLT); ultrasound (US) therapy; and physical therapeutic exercises (PTEs), which consisted of a 40 min session with moderate-intensity exercises, including a 5 min warm-up on a stationary bike, static quads with a 7 s hold, knee extensions over a roll with a 7 s hold, single-leg raises for 50 repetitions, step-ups for 50 repetitions, calf raises for three sets of 10–15 repetitions, and wall squats with a 5–10 s hold [15]. Also, neuroproprioceptive facilitation (PNF) techniques were incorporated into four movement patterns, and cryotherapy in the form of ice packs was applied at the conclusion of the physiotherapy session to cool down the affected knee [15].

The study observed that the WOMAC score, which assesses osteoarthritis severity, decreased for both groups of patients, but notably, the treated group showed significant improvements at the 3-month mark. This led to the conclusion that physical therapy consistently enhanced the quality of life for these patients. Additionally, it was demonstrated that VAS pain scores decreased from 5.7 to 2 in the treated group. Furthermore, the treated group experienced an increase in muscle strength [15].

Another multicenter study was conducted to assess the effectiveness of a comprehensive approach for treating and rehabilitating KOA. This approach combined physical therapy with HA injections, aiming to provide both clinical benefits and cost-effectiveness [78]. The rehabilitation program featured injectable HA and therapeutic physical exercises, including muscle strengthening, flexibility routines, and proprioception training [78].

Specifically, the study involved 553 patients with symptomatic KOA who had previously undergone unsuccessful pharmacological treatments. These patients were monitored over an 8-week period at 27 specialized centers across the USA. Guided by experienced physiotherapists, they participated in physical therapy sessions two to three times per week.

The results were promising, showing a significant reduction in knee pain, with a 59% decrease in all patients. The WOMAC score improved by 44% to 51%. Even patients with advanced stages of KOA (KL3–KL4) experienced a notable reduction in specific symptoms [78].

Moreover, the combined therapy program proved to be cost-effective over a 2-year monitoring period. It postponed the need for surgical knee treatments, underscoring the potential of rehabilitation through physical therapy and HA injections to slow down KOA progression while maintaining a favorable cost-effectiveness ratio [78].

Long-term outcomes measured included knee pain severity, medication use, knee operations, and health utility scores. The results were promising, with a reduction in knee pain severity during the 8-week program. At 1 and 2 years post-treatment, medication use remained common, and the utilization rates for total knee arthroplasty were 10.4% and 18.0%, respectively. Additionally, health utility scores improved significantly. This study demonstrated that the 8-week multimodal KOA treatment program provided meaningful improvements in KOA symptoms, even in advanced cases, and was cost-effective over a 2-year follow-up period [78].

A recent postmarket, single-blind, multicenter randomized controlled clinical trial was conducted to evaluate the treatment outcomes in a relatively young and active population of individuals with patellofemoral OA and/or tibiofemoral OA (NCT03281837). The trial aimed to compare the responses to two weekly intra-articular HA injections, administered with a 1-week interval between injections, of HYMOVIS 24 mg/3 mL (Fidia Pharma USA Inc., Florham Park, NJ, USA) in combination with a physical exercise program versus a physical exercise program alone (NCT03281837). In this study, 148 patients were divided into three groups: the first group received one injection of Hymovis per week for two consecutive weeks, along with at least 8 weeks of a physical exercise program; the second group underwent treatment with only the physical therapy program, without any additional intervention, while the last group included patients who were randomized to receive only the physical exercise program initially. If they did not respond to this intervention after 3 months, they had the option to cross over and receive two intra-articular weekly injections of Hymovis, with each injection given 1 week apart (NCT03281837). As of now, the results of this study have not been published, but it is hypothesized that the combined therapy approach, involving HA injections and physical exercise, will demonstrate its effectiveness in managing patellofemoral and tibiofemoral osteoarthritis (NCT03281837).

A very recent study conducted by Ma et al. (2023) compared the effectiveness of two treatment approaches for KOA, one involving leg swinging and quadriceps strengthening exercises and the other involving a combination therapy of platelet-rich plasma (PRP) and HA. This trial included 106 patients with KOA graded as I–III according to the KL scale, who were divided into two groups [79].

The first group underwent a regimen of leg swinging exercises and quadriceps strengthening exercises for a duration of 3 months. Specifically, the leg swinging exercise involved patients placing their unaffected leg either on the floor or the edge of a platform, allowing the affected leg to swing freely in the air. To maintain balance and prevent falls, patients were permitted to use one hand to support themselves against a wall or railing. Patients were instructed to initially lift their leg to approximately 45° from the vertical line and then gradually increase the range to around 60°. Patients were encouraged to perform approximately 500 leg swings daily [79].

For the quadriceps strengthening exercise, patients were directed to keep the ankle joint of the affected leg in a neutral position with plantar flexion, extend the knee joint to 0°, and slowly raise the leg until the heel was 25 to 30 cm above the bed. They were instructed to hold this position for 5 to 10 s and then gradually lower the leg to a supine position. Patients were advised to repeat this exercise approximately 200 times per day. These two physical exercises were to be alternated during the patient’s spare time, with enrolled patients instructed to perform them daily at home for a period of 3 months [79].

The second group of patients received intra-articular injections of 2 mL each of PRP and HA (Eufflexa, Ferring Pharmaceuticals, Saint-Prex, Suisse), with the HA having a high MW ranging from 2.4 to 3.6 million Da. These injections were administered every 2 weeks [79].

It was demonstrated that both groups showed improvements in pain, quality of life, balance ability, and functional activity [79]. However, the leg swing and quadriceps strengthening exercise group exhibited more significant improvements compared with the intra-articular PRP combined with HA injection group, and these benefits were sustained even after 6 months [79].

In addition to KOA, HA has also demonstrated effectiveness in the treatment of glenohumeral OA. This condition is characterized by degenerative changes in the cartilage, synovial membrane, synovial fluid, and subchondral bone of the shoulder joint [80,81]. As a result, individuals with glenohumeral OA experience persistent shoulder pain and a reduced range of motion (ROM) [80,81]. In this case as well, the therapeutic approach involved intra-articular injections of HA or corticosteroids, physical therapy, and the use of oral analgesics [82,83,84,85,86].

However, it is worth noting that the use of oral analgesics can lead to the occurrence of side effects [87,88].

A randomized controlled prospective open-label monocentric study conducted by Di Giacomo and De Gasperis (2017) demonstrated the effectiveness of intra-articular injections of HA in combination with a specialized physiotherapy program for the treatment of shoulder OA [89]. This study enrolled 78 patients who were affected by grade II and III OA, as well as grade IV shoulder OA, and were not candidates for surgical treatment due to contraindications [89]. The patients were randomly divided into two groups. The treated group received three intra-articular injections of high MW HA (>1500 MDa) (Hyalubrix, 30 mg/2 mL, Fidia Farmaceutici S.p.A., Abano Terme, Italy), with one injection administered every 15 days. This was combined with a specific physiotherapy program. In contrast, the control group received treatment solely through physical therapy [89].

The physiotherapy program had a duration of 3 months, with sessions conducted 3 days a week. It commenced for both groups the day following the initial medical examination. The program encompassed passive capsular stretching to restore the ROM, isometric exercises targeting the deltoid, rotator cuff, and scapulothoracic muscles, isotonic exercises for the scapulothoracic muscles (closed kinetic chain), and hydrokinesis therapy [89].

Results showed that patients who received the combined treatment experienced a more significant reduction in pain compared with those who underwent only physical therapy. Furthermore, the reduction in pain led to improvements in glenohumeral function and the ability to carry out daily life activities [89].

In a 2015 open-label study conducted by Di Giacomo and De Gasperis, they examined the impact of a treatment regimen that combined intra-articular HA injections with a specific physical therapy program in 61 patients diagnosed with shoulder OA at stages I, II, or III. The primary focus of this investigation was to evaluate the reduction in shoulder pain and improvements in ROM [90]. All participants in the study experienced shoulder pain with varying degrees of severity, ranging from mild to moderate and severe.

Similar to the earlier study, the patients in this trial were divided into two groups. The first group received five intra-articular injections of Hyalgan (Fidia Farmaceutici, Abano Terme, Italy), each containing 20 mg/2 mL (with a MW of 500–730 kDa). They also followed a specific physiotherapy program. Conversely, the second group solely underwent physical therapy [90].

The physiotherapy program, in this case, was supervised by a professional therapist and had a duration of 3 months, with sessions held 3 days per week. The program included passive capsular stretching to restore ROM, isometric exercises targeting the deltoid, rotator cuff, and scapulothoracic muscles, isotonic exercises for the scapulothoracic muscles in a closed kinetic chain, and hydrokinesis therapy [90].

The results of the study clearly indicated a significant reduction in shoulder pain in both groups that received the two different treatments. Furthermore, it underscored a substantial difference in the degree of shoulder pain between the two groups of patients. These findings support the notion that patients treated with intra-articular HA injections combined with physical therapy experience a more substantial and enduring positive effect compared with those who solely receive physical therapy [90].

Similarly, the study revealed a significant enhancement in forward elevation in both groups, with the improvement being notably more pronounced when comparing the two groups. Once again, this underscores that patients who underwent intra-articular HA injections and physical therapy derived greater benefits in terms of ROM improvement in forward elevation. This improvement could be attributed to the significant reduction in shoulder pain experienced by these patients [90].

In summary, this study provided compelling evidence that intra-articular HA injections were beneficial for treating patients with shoulder OA. These injections resulted in a significant reduction in shoulder pain and a partial recovery of ROM, leading to improved daily activities. This positive effect can be attributed to the specific properties of Hyalgan, which has the capacity to restore synovial fluid properties in this disease [75,90,91,92,93,94].

#### HA-Based Therapy in Low Back Pain

It is important to note that OA can manifest various symptoms, including lower back pain (LBP) [12], which is a highly prevalent issue. It is acknowledged that several underlying factors can contribute to LBP, encompassing conditions such as tumors, infections, fractures, and inflammatory disorders. Nonetheless, OA stands out as the most common cause. Additionally, mechanical and structural issues, such as lumbar spinal stenosis, spondylolysis, spondylolisthesis, and congenital deformities like scoliosis or hyperkyphosis, can contribute to LBP [12].

Current guidelines advocate exercise as the primary approach for addressing LBP [95,96]. Remarkably, rehabilitation utilizing the McKenzie method appears to yield superior short-term pain reduction and disability management in both acute and chronic LBP when compared with other physiotherapeutic approaches. A recent study demonstrated the effectiveness of oral viscosupplementation, involving a blend based on Fortigel (Gelita, Eberbach, Germany), including collagen peptides, Vitamin C, copper, manganese, and sodium hyaluronate, in conjunction with the McKenzie method kinesitherapy, compared with kinesitherapy alone in the treatment of chronic LBP due to osteoarthritis [12].

Specifically, in this randomized clinical trial, 60 patients were randomly allocated to two groups. Both groups underwent physiatric evaluations, encompassing medical history, physical exams, and imaging. Group A received a 3-week McKenzie rehabilitation program consisting of nine sessions, while group B received the same program in addition to a daily dietary supplement of Fortigel, which contains vitamin C (80 mg), sodium hyaluronate (50 mg, with 46 mg as HA), manganese (1 mg), and copper (0.5 mg), throughout the treatment period [12].

The findings indicated that both group A and group B experienced improvements in pain and disability scores (VAS and Oswestry Disability Index, ODI). Particularly, group B demonstrated a more significant reduction in VAS and ODI scores, and these scores remained relatively stable. It was also observed that both groups experienced an increase in the Short Form-12 (SF-12) physical dimension (PCS-12) score. However, even in this case, group B’s score remained stable [12].

This suggests that a combined treatment approach involving McKenzie back rehabilitation and oral supplementation with collagen peptides, HA, vitamin C, manganese, and copper can effectively reduce pain and motor disability and enhance the quality of life of patients suffering from chronic LBP due to OA [12].

In conclusion, these studies collectively demonstrate the effectiveness of HA-based products (injectables or oral-based formulations) when combined with physical and rehabilitative interventions in the treatment of OA and symptoms associated with it.

In this way, HA can alleviate pain and improve joint function, while physical therapy and rehabilitative exercises can further augment their effectiveness by strengthening the muscles around the affected joint, enhancing ROM, and supporting overall joint health (Table 1).

### 3.2. HA-Based Therapy in Tendinopathies Treatment

Tendinopathies are a group of conditions that can affect tendons, and they can involve various processes, including inflammation, degeneration, or lesions of the tendon [97]. 

Tendon disorders are very common, and they often have a negative impact on patients’ quality of life [11,97]. The etiology of these conditions involves a multitude of factors, including mechanical overload, reduced blood flow, age, gender, and genetic, hormonal, and metabolic components [98,99,100,101]. Tendinopathic tendons display widespread structural alterations, such as increased tenocyte cell death, disruption of collagen fibers resulting in reduced production of collagen type I, an abnormal surge in type III collagen production, and ineffective formation of new blood vessels [98,102].

Patients commonly report pain localized at the affected tendon site, which intensifies during physical activity and daily life [103]. Treatment approaches for tendinopathy are a subject of ongoing debate; however, HA has been explored as a potential treatment option for some tendinopathies, particularly those involving inflammation and pain, since it is useful in tendon regeneration [104,105,106].

Specifically, tendon lesions are prevalent in sportsmen and physical workers [107], and they can manifest as either complete or partial lesions. Notably, there has been emerging research suggesting the potential of HA for treating such conditions attributed to its anti-inflammatory and lubricating properties.

In a prospective, open-label, multicenter clinical study led by Frizziero et al. (2019), 35 patients with symptomatic Achilles or patellar midportion tendinopathy were enrolled to assess the effectiveness of medium MW sodium hyaluronate (500–730 KDa) at a concentration of 20 mg/mL (Hyalotend, Fidia Farmaceutici, Abano Terme, Italy) [108].

The treatment involved administering HA via peritendinous ultrasound-guided injections placed between the paratenon and the tendon. Over the course of three consecutive weeks, each patient received a 2 mL injection weekly. The evaluation of outcomes occurred at the 90-day follow-up [108].

To assess functional improvement, Italian versions of the Victorian Institute of Sports Assessment-Achilles’ questionnaire (VISA-A) and the Victorian Institute of Sports Assessment-Patellar (VISA-P) were employed. The mean change from baseline in the VISA-A and VISA-P total scores was calculated, utilizing either the prevalence or the last observation carried forward (LOCF) approach [108].

In addition to these assessments, the study examined changes in pain using the Numeric Pain Rating Scale-11 (NRS-11), clinical parameters such as redness, warmth, swelling, tenderness on palpation, crepitus on motion, and accumulation of tissue fluid, as well as improvements in ultrasound parameters like the axial and sagittal thickness of the target tendon and neovascularization assessed via power Doppler using a 4-point scale. Other evaluations included the Patient Global Assessment (PGA), Clinical Observer Global Assessment (COGA), consumption of rescue medication (paracetamol), and assessment of Health-related Quality of Life using the EuroQoL EQ-5D-5L questionnaire [108].

The study concluded that the treatment was generally well-tolerated, with only one adverse event reported in the Achilles tendinopathy group, which was likely related to the injection procedure [108].

Total tendon lesions, where the tendon is completely severed, often require more invasive treatment compared with partial injuries, which is why HA is less commonly used in these cases.

Indeed, HA has shown its effectiveness in addressing even the most severe tendon disorders. A recent clinical trial, which has not yet published its results, aimed to compare the effectiveness of steroid, HA, platelet-rich plasma PRP, and a placebo (normal saline) in treating partial rotator cuff tears. This study also incorporated the same physical therapy protocol after injection (NCT04681937).

The trial likely followed a randomized controlled design, where 80 participants were divided into four groups: the first group received a subacromial injection of sodium hyaluronate (4 mL), followed by the same physical therapy regimen applied to all groups after injection; the second group received a subacromial injection of platelet-rich plasma (PRP) (4 mL) and underwent the same physical therapy; the third group received subacromial injections of methylprednisolone acetate (1 mL) along with the same physical therapy; and the last group received a placebo injection and followed the same physical therapy protocol (NCT04681937).

The objective of this study was to assess and compare several outcome measures, including the American Shoulder and Elbow Surgeons Shoulder Score (ASES), the Constant–Murley Shoulder Outcome Score (CMS), VAS for pain assessment, Subjective Shoulder Value (SSV), and ROM (NCT04681937).

Another recent prospective nonrandomized comparative study conducted by Huang et al. (2022) investigated the effectiveness of PRP and HA injections for the treatment of partial-thickness rotator cuff tears. Specifically, the study aimed to compare the outcomes of ultrasound-guided single PRP injection with three doses of HA injection (Hyruan Plus, LG Pharm Co., Ltd., Seoul, Korea), combined with postinjection rehabilitation, for managing partial-thickness rotator cuff tears. The study enrolled 48 patients who were divided into two groups: 24 patients received ultrasound-guided PRP intralesional and peritendinous injections along with rehabilitation exercises, while the remaining 24 patients received three doses of HA subacromial injections in addition to rehabilitation exercises [109].

In this case, therapeutic exercises included shoulder ROM exercises, flexibility exercises, scapular stabilization exercises, and shoulder girdle strengthening exercises [109].

The study assessed outcomes using the Shoulder Pain and Disability Index (SPADI), ROM measurements, VAS scores, and the Constant–Murley Shoulder Score (CMSS). These measurements were taken before the injection and at 1 and 3 months after the injection [109].

The results indicated that in the PRP group, SPADI scores, VAS scores, and CMSS showed significant improvements at both the 1-month and 3-month follow-ups. Furthermore, flexion and abduction ROM significantly increased at the 3-month follow-up. In the HA group, SPADI scores, VAS scores during overhead activities, VAS night pain, and CMSS also showed significant improvements at both the 1-month and 3-month follow-ups, with flexion and active abduction ROM significantly increasing in the third month [109].

These findings suggest that both PRP and HA injections, when combined with rehabilitation exercises, can effectively improve outcomes for patients with partial-thickness rotator cuff tears. However, the study emphasizes the need for further research to confirm these results and explore the long-term effects of these treatments [109].

In a study conducted by Flores et al. (2017), the efficacy and safety of peritendinous HA injections were investigated in patients with persistent supraspinatus tendinopathy. This study employed a parallel-group randomized controlled trial design [110]. Specifically, the researchers aimed to compare the therapeutic outcomes of treatment with HA as an adjuvant to physical therapy with those of physical therapy as the sole therapeutic intervention [110].

A total of 84 patients were randomly assigned to two study groups: the HA group received treatment with physical therapy in conjunction with a subacromial injection of HA (40 mg sodium hyaluronate/2 mL, MW 1.6 MDa) using OSTENIL TENDON (TRB CHEMEDICA AG, Feldkirchen/Munich, Germany), while the control group underwent physical therapy alone [110].

To assess treatment efficacy, the researchers used a VAS for pain and an Activities of Daily Living (ADL) scale. Other measures included the number of rehabilitation sessions required and the days needed for recovery, the Tampa Scale for Kinesiophobia (TSK), and the perception of efficacy and tolerability by both the physician and the patients. The patients were followed up for 90 days [110].

Overall, both the VAS and ADL scores exhibited a progressive decrease during the follow-up period, with no significant differences between the two groups. However, the TSK score showed a significant decrease in the HA group compared with the control group. Additionally, patients in the control group required more rehabilitation sessions and more days to return to their preinjury activity levels. Both patients and investigators perceived higher efficacy in the HA group than in the control group. Importantly, both treatments were found to be safe and well-tolerated [110].

This study demonstrated that subacromial HA injections, when combined with physical therapy, had a high efficacy in the treatment of supraspinatus tendinopathy. This approach led to an earlier return to preinjury activity levels and reduced the need for extensive rehabilitation sessions, which could be beneficial both for patients and the healthcare system [110].

In another preliminary open-label study, a total of 61 patients with varying tendon-related conditions were enrolled, including 14 with patellar tendinopathy [111]. The aim of the study was to demonstrate the effectiveness of high-molecular-weight HA (Suvenyl, Chugai Pharmaceutical Co., Ltd., Tokyo, Japan) [111].

During the study, patients received a single injection of HA, with a maximum volume of 2.5 mL, targeted at the attachment site of the affected tendon or ligament. In the case of patellar tendinopathy, HA was precisely injected into the proximal interface between the posterior surface of the patellar tendon and the infrapatellar fat pad [111]. This procedure was performed with the patient’s knee extended, and the needle was carefully inserted to reach the interface between the patellar tendon and the infrapatellar fat pad [111].

The outcomes of this trial revealed that a single HA injection had a significant positive impact on patellar tendinopathy. Notably, 50% of the patients with patellar tendinopathy experienced a substantial improvement of at least 50% in their VAS pain scores after receiving the injection compared with their baseline scores. This promising result suggests that HA could be a viable treatment option for individuals with patellar tendinopathy [111].

These recent trials have investigated the potential of HA as a viable treatment option for tendinopathies, especially those connected with inflammation and pain. The combined findings from these studies indicate that HA injections, whether administered on their own or in conjunction with suitable rehabilitation approaches, hold promise as effective treatment choices for a range of tendinopathies. In summary, HA appears to be a valuable addition to existing treatments for tendinopathies, potentially leading to better outcomes and an improved quality of life for individuals affected by these conditions (Table 2).

### 3.3. HA-Based Therapy in Meniscal Lesions

The meniscus is a fibrocartilaginous disk-like structure located within the knee joint. An undamaged meniscus serves several crucial functions within the joint, including stabilizing the joint, distributing the load across the joint surface, absorbing shocks, providing lubrication, and facilitating nutrient supply [112,113].

Meniscal injuries, which are the second most common type of knee injury, carry a significant risk due to their potential to cause joint instability and reduced impact resistance, increasing the likelihood of developing degenerative osteoarthritis [114].

In particular, meniscal tears can be classified into two main types: traumatic and degenerative. Traumatic tears typically result from acute injuries or trauma and are more commonly observed in younger patients. In contrast, degenerative tears are more prevalent among older patients and typically occur due to gradual intrasubstance degeneration within the menisci over time [115]. Traditional treatments include physical therapy, pharmacologic approaches (including paracetamol, nonsteroidal anti-inflammatory drugs, and intra-articular corticosteroid injections) [116,117,118,119,120], and surgical interventions. However, there is ongoing research exploring the potential therapeutic role of HA in managing meniscal injuries [121].

A recent randomized clinical trial conducted by Başar et al. (2021) compared the effectiveness of two treatment approaches for patients with symptomatic degenerative meniscus tears: arthroscopic partial meniscectomy (APM) and physical therapy. The study also investigated the impact of HA injections on the outcomes of these treatments [17].

The trial included a total of 192 patients, who were randomly assigned to one of four groups. The first group received APM alone, the second group underwent APM followed by HA injections, the third group received only physical therapy, and the fourth group underwent physical therapy combined with HA injections. The physical therapy regimen in this study involved the use of TENS and low-intensity pulsed ultrasound [17].

During the exercise therapy, a program consisting of 104 progressive neuromuscular and strength exercises was administered. These exercises were conducted three times a week for 4 weeks initially and then continued for an additional 8 weeks, also with a frequency of three sessions per week. The exercise routine included single-leg strength training for both the injured and uninjured sides, encompassing both concentric and eccentric movements in positions with and without weight bearing. The program began with two sets of 15 repetitions and gradually progressed to three sets of 12 repetitions, followed by three sets of 8 repetitions, and concluded with four sets of 6 repetitions [17].

Regarding the HA injection, high MW HA was used. In the second group, HA injections were administered as a single injection 4 weeks after the APM procedure, while in the fourth group, HA injections were given as a single injection before the commencement of physical therapy [17].

At the end of this study, it was observed that both the WOMAC and VAS scores had improved compared with pretreatment values at the end of the second and sixth months. However, there were no significant differences in WOMAC and VAS scores between the four treatment groups. Notably, the APM groups exhibited relatively worse results.

Additionally, APM combined with physical therapy yielded positive outcomes in terms of pain relief and functional improvements, although there were limitations in ROM following APM. Conversely, physical therapy led to an increase in ROM. Interestingly, the presence of HA injections did not appear to significantly influence these results [17].

In contrast, in a recent noninterventional prospective multicenter study conducted by Balius et al. (2023), 165 patients were included, with 58 of them suffering from degenerative knee meniscal tear. These patients received two consecutive intra-articular injections of a noncross-linked, partially hydrophobized derivative of HA (Hymovis, Fidia Farmaceutici Abano Terme, Italy) at a 2-week interval [121].

The study revealed significant improvements in the patients’ overall quality of life (QoL), which was assessed using various measures, including the Knee Injury and Osteoarthritis Outcome Score (KOOS), WOMAC score, Patient’s Global Improvement Impression Scale (PGI-I), and a single question [121]. Specifically, improvements in physical activity were assessed through activities of daily life and sports and recreation participation.

The results showed that 80% of patients reported a global improvement in their QoL after the treatment, with only 6.8% indicating no change or impairment [121].

Furthermore, there were statistically significant improvements in all individual dimensions of KOOS and WOMAC, with a substantial increase (28%) in activities of daily living. A significant 95.6% of patients perceived significant improvement after the treatment, and both VAS satisfaction and pain assessment improved [121].

Importantly, no serious adverse effects related to Hymovis or adverse reactions were observed in the study.

The efficacy of Hymovis in treating degenerative meniscus lesions was also investigated [122]. The trial included forty patients with degenerative meniscus lesions confirmed via MRI. These patients received two injections of Hymovis (HYADD4, a noncrosslinked HA alkylamide, 24 mg/3 mL), spaced 2 weeks apart [122]. The study revealed notable improvements in both the WOMAC score and the physical function subscale following the treatment. Furthermore, assessments such as PGA and CoGA showed consistent improvement over time. These findings collectively demonstrate the effectiveness of Hymovis (Fidia Farmaceutici, Abano Terme, Italy) in promoting meniscus healing [122].

Therefore, these research findings emphasize the significance of exploring alternative treatments such as HA injections as a viable option for managing meniscal injuries, offering an alternative to surgical interventions. These insights offer valuable perspectives on the changing field of meniscus injury care, underscoring the necessity for customized approaches that cater to the unique requirements of patients dealing with meniscal lesions (Table 3).

## 4. Conclusions

HA stands out as an important biocompatible and biodegradable compound with substantial potential as a bioactive molecule for addressing a wide spectrum of both physiological and pathological conditions, with a particular focus on musculoskeletal rehabilitation. Its significance stems from its abundance in connective tissues, particularly within joints. In fact, within the synovial fluid, HA plays an essential role by lubricating joints, effectively mitigating friction, and contributing significantly to overall joint health. Additionally, HA’s distinctive attributes, including its viscosity and water retention capabilities, make it an invaluable component for enhancing flexibility and absorbing shocks during various types of movement.

This review article primarily provided an in-depth exploration of HA’s functions and properties. It is evident that these properties collectively establish HA as an exceptionally effective therapeutic compound for a wide range of medical conditions, especially those related to musculoskeletal diseases, and as a senomorphic agent [123]. Consequently, several HA-based products have been developed and are readily accessible in the market.

Furthermore, the article discussed the applications and effectiveness of HA, either on its own or in combination with physical therapy, in the most common joint diseases, including OA, tendinopathies, and meniscal injuries. The findings suggest that HA, whether used alone or in conjunction with physical therapy, offers a functional approach. In this manner, the synergy of HA can alleviate pain and improve joint functions in OA treatment, reduce pain and inflammation in tendinopathies, and even facilitate the repair of meniscal lesions. Contemporary applications of physical therapy and rehabilitative exercises further enhance the effectiveness of these treatments by strengthening the muscles or the affected joint.

In conclusion, the advancement and application of innovative HA-based products hold great significance as a promising avenue for addressing musculoskeletal disorders. Additionally, it remains essential to persist in researching and evaluating the impact of combined therapies involving HA and physical therapy. This ongoing exploration holds the potential to further enhance our understanding and improve treatment outcomes for individuals with musculoskeletal conditions.

This study offers a detailed and thorough examination of the most recent scientific evidence regarding the biochemical and clinical dimensions of HA effectiveness in the rehabilitation of musculoskeletal diseases. As outlined, these topics are too expansive to be adequately covered in a single systematic review. Consequently, further studies are needed to focus on a more targeted exploration of each theme, employing individualized and specific systematic reviews and meta-analyses.

## Figures and Tables

**Table 1:** HA-based therapy in OA treatment.

Condition	HA-Based Product	HA Molecular Weight	Physical Therapy	Method	Result	Follow-Up Period	Ref.
KOA	Orthovisc (Anika Therapeutics Inc., Bedford, MA, USA)	1.0–2.9 MDa	Detailed program of knee exercises for a total of 20 treatment sessions in a month	Blinded prospective randomized controlled study	Improvements in pain, stiffness, and function	1 month	[13]
KOA	Kombihylan (Ropharm, Romania)	3 MDa	Electrotherapy, TENS, LLLT, US, PTE, PNF	Monocentric observational study	Decrease in WOMAC and VAS scores, increase in muscle strength	12 months	[15]
KOA	Not described	Not described	Muscle strengthening, proprioception, and flexibility exercises; knee bracing	Multimodal treatment program	Reduction in knee pain and specific symptoms	24 months	[78]
Patellofemoral OA and/or tibiofemoral OA	HYMOVIS, (Fidia Pharma USA Inc., Florham Park, NJ, USA)	Crosslinked HA, MW not computable	Physical exercise program	Postmarket, single-blind, multicenter randomized controlled trial	unposted	3 and 6 months	[NCT03281837]
KOA	Eufflexa (Ferring Pharmaceuticals, Saint-Prex, Suisse)	2.4–3.6 MDa	Leg swinging and quadriceps strengthening exercises	Retrospective comparative study	Improvements in pain, QoL, balance ability, and functional activity	12 months	[79]
Shoulder OA	Hyalubrix (Fidia Farmaceutici S.p.A., Abano Terme, Italy) and Hyalgan (Fidia Farmaceutici, Abano Terme, Italy)	1.5 MDa	Capsular stretching, strengthening the deltoid, rotator cuff, and scapulothoracic muscles with isometric exercises, isotonic exercises for scapulothoracic muscles, and hydrokinesis therapy	Prospective randomized study	Reduction in pain, improvements in glomerular functions, ROM, and QoL	6 months and 18 months	[89,90]
LBP caused by OA	Fortigel (Gelita, Eberbach, Germany)	Not described	McKenzie method kinesitherapy	Randomized clinical trial	Reduction in VAS and ODI scores	6 weeks	[12]

**Table 2:** HA-based therapy in tendinopathies treatment.

Condition	HA-Based Product	HA Molecular Weight	Physical Therapy	Method	Result	Follow-Up Period	Ref.
Achilles tendinopathy	Hyalotend (Fidia Farmaceutici, Abano Terme, Italy)	Medium MW		Prospective multicentric clinical trial	Reduction in pain	90 days	[108]
Rotator cuff tears	Not described	Not described	Physical therapy procedure after injection	Prospective randomized study	Unposted	Not described	NCT04681937
Partial-thickness rotator cuff tears	Hyruan Plus (LG Pharm Co., Ltd., Seoul, Korea)	3 MDa	Shoulder ROM, flexibility, scapular stabilization exercise, and shoulder girdle strengthening exercise	Prospective nonrandomized comparative study	Improvements in SPADI, VAS during overhead activities, VAS night pain, and CMSS scores	1 and 3 months	[109]
Supraspinatus tendinopathy	OSTENIL TENDON (TRB CHEMEDICA AG, Feldkirchen/Munich, Germany)	1.6 MDa	Pendulum exercise, scapular retraction, posterior capsule stretch, trapezius stretch, internal and external rotation, row, lower trapezius	Multicenter randomized controlled trial	Decrease in VAS, TSK, and ADL scores	90 days	[110]
Patellar tendinopathy	Suvenyl (Chugai Pharmaceutical Co., Ltd., Tokyo, Japan)	2.7 MDa		Prospective open-label preliminary study	Improvement in VAS pain scores	1 week	[111]

**Table 3:** HA-based therapy in meniscal lesions treatment.

Condition	HA-Based Product	HA MW	Physical Therapy	Method	Result	Follow-Up Period	Ref.
Degenerative meniscus tears	High MW HA	Not described	TENS, low-intensity pulsed ultrasound, single-leg strength training (concentric and eccentric movements)	Randomized clinical trial	Improvement in WOMAC and VAS scores (the presence of HA seems to not influence these results)	5 years	[17]
Degenerative knee meniscal tear	Hymovis (Fidia Farmaceutici Abano Terme, Italy)	Crosslinked HA, MW not computable		Prospective, noninterventional, post-marketing, observational, descriptive multicenter study	Improvements in QoL, physical activity, KOOS, and WOMAC scores	7 months	[121]
Degenerative meniscus lesions	Hymovis (Fidia Farmaceutici Abano Terme, Italy)	Crosslinked HA, MW not computable		Open-label prospective pilot study	Improvements in WOMAC score, physical function, PtGA, and CoGA	1 year	[122]

## Data Availability

Not applicable.
